# 

**Published:** 1904-12

**Authors:** 


					﻿Society Notes.
Smith County Medical Society, annual meeting, Tyler,
Texas, December 13, inst.' Dr. Irvin Pope, President; Dr. A. Wol-
dert, Secretary.
Van Zandt County Medical Society met at Grand Saline
December 2d, inst. Dr. T. A. Martin, President; Dr. 0. M. March-
man, Secretary.
Southern Surgical and Gynecological Association, seven-
teenth annual meeting, Birmingham, Ala., December 13, 14, and
15th inst. Dr. F. W. McRae, President, Atlanta, Ga.
' The North Texas Medical Association, a very large organi-
zation, will meet at Paris, Texas, December 13th and 14th. Dr.
H. L. Moore, Van Alstyne, Secretary. Usual rates on all roads.
The Pan-American Medical Congress will be held at Panama,
.January 2-6 (prox.). Special rates on ship for delegates. Write to
Prof. R. Matas, New Orleans, for particulars. The President of
the State Medical Association has appointed the following dele-
gates: Drs. H. K. Leake and J. 0. McReynolds, Dallas; Dr. Geo.
H. Lee, Galveston; Dr. A. E. Spohn, Corpus Christi.
To attend a dead-beat whelp, was I away;
Opportunity called, a kindly word to say;
Priceless pearls before stupid swine;
Thus to work is worse than wasted time;
And this is why I am poor, at the age .of sixty-four.
November, 1904,	Q. C. Smith, M. D.
				

